# Pertussis surveillance and control: exploring variations and delays in testing, laboratory diagnostics and public health service notifications, the Netherlands, 2010 to 2013

**DOI:** 10.2807/1560-7917.ES.2017.22.28.30571

**Published:** 2017-07-13

**Authors:** Jeanne Heil, Henriëtte L G ter Waarbeek, Christian J P A Hoebe, Peter H A Jacobs, Dirk W van Dam, Thera A M Trienekens, Jochen W L Cals, Inge H M van Loo, Nicole H T M Dukers-Muijrers

**Affiliations:** 1 Department of Sexual Health, Infectious Diseases and Environmental Health, South Limburg Public Health Service, Geleen, the Netherlands; 2 Department of Medical Microbiology, School of Public Health and Primary Care (CAPHRI), Maastricht University Medical Centre (MUMC+), Maastricht, the Netherlands; 3 Department of Infectious Diseases Control, North Limburg Public Health Service, Venlo, the Netherlands; 4 Department of Medical Microbiology and Infection Prevention, Zuyderland Medical Centre, Sittard-Geleen, the Netherlands; 5 Department of Medical Microbiology, VieCuri Medical Centre, Venlo, the Netherlands; 6 Department of General Practice, School of Public Health and Primary Care (CAPHRI), Maastricht University Medical Centre (MUMC+), Maastricht, the Netherlands

**Keywords:** Pertussis, vaccine-preventable disease, surveillance, laboratory, general practitioners, infectious disease control

## Abstract

Pertussis is most severe among unvaccinated infants (< 1 year of age), and still leads to several reported deaths in the Netherlands every year. In order to avoid pertussis-related infant morbidity and mortality, pertussis surveillance data are used to guide pertussis control measures. However, more insight into the accuracy of pertussis surveillance and control, and into the range of healthcare and public health-related factors that impede this are needed. We analysed a unique combination of data sources from one Dutch region of 1.1 million residents, including data from laboratory databases and local public health notifications between 2010 and 2013. This large study (n = 12,090 pertussis tests) reveals possible misdiagnoses, substantial under-notification (18%, 412/2,301 laboratory positive episodes) and a delay between patient symptoms and notification to the local public health services (median 34 days, interquartile range (IQR): 27–54). It is likely that the misdiagnoses, under-notification and overall delay in surveillance data are not unique to this area of the Netherlands, and are generalisable to other countries in Europe. In addition to preventive measures such as maternal immunisation, based on current findings, we further recommend greater adherence to testing guidelines, standardisation of test interpretation guidelines, use of automatic notification systems and earlier preventive measures.

## Introduction

*Bordetella pertussis* and *Bordetella parapertussis* infections are most severe among unvaccinated infants [1-4]. Complications of the resulting disease, pertussis, include pneumonia, failure to thrive from post-tussive vomiting, seizures, secondary bacterial infection and pulmonary hypertension [2,5]. The full implementation of general pertussis vaccination in the 1950s greatly reduced its incidence in the Netherlands [6] and led to a shift from cases in children to adults [5,7-9]. Even though worldwide vaccination coverage of 86% has been achieved [10], there were around 63,000 pertussis-linked deaths in children under 5 years of age in 2012 [11]. In Europe, the highest number of cases were notified by the Netherlands in 2012 (n = 12,868), accounting for 30% of all notifications in Europe [12]. The Dutch incidence rate of symptomatic pertussis infections in 2011 was estimated to be 107 per 10,000 population [13].

The Netherlands has an extensive free of charge National Immunisation Programme (NIP) to protect all children against 12 infectious diseases, including pertussis [14]. The efficacy of pertussis vaccines has been debated because of waning immunity, incomplete protection of infants younger than 5 months of age, genetic changes in *B. pertussis* and limited duration of protection [3,15,16]. Maternal immunisation is recommended by the WHO, ECDC and by the Health Council of the Netherlands [12,17,18]. In England, maternal immunisation was found to have a vaccine-effectiveness of 91% in infants < 2–3 months of age with no side effects [19,20]. Despite a pertussis vaccination coverage in the Netherlands of 96%, increasing numbers of pertussis notifications have been observed since 1996 [15,21,22], with epidemic peaks every 2–3 years [15] and 1 to 3 pertussis-related deaths per year [14].

Pertussis is a notifiable disease in the Netherlands according to Wet Publieke Gezondheid, the law that requires notification of NIP-targeted diseases [23]. Local public health services (PHS) must be notified when: (i) patients have typical symptom(s) or (ii) patients have at least 14 days of coughing, combined with either a positive laboratory test or recent contact with a confirmed pertussis case [24]. Legally, both healthcare providers (HCPs) and laboratories are responsible for notification, but in practice, most notifications originate from the laboratories. Local PHS collect and verify all notifications, advise patients’ HCPs on vaccination and/or medical treatment of contacts, and report cases to the National Institute for Public Health and the Environment (RIVM). An overview of the guidelines and criteria for pertussis testing, diagnostics and notifications in the Netherlands is provided in Table 1.

**Table 1:** Pertussis testing, diagnostics and notification guidelines and criteria, the Netherlands, 2010–2013

Actor	Responsibility	Guidelines/criteria
**Healthcare provider**	Clinical diagnostic	Patients with typical symptoms^a^ or, during epidemics, patients with severe coughing who have had contact with a proven pertussis case [45].
	Requests for laboratory testing	When pertussis is suspected in a patient whose family includes unvaccinated or incompletely vaccinated infants < 1 year of age or a woman > 34 weeks pregnant [45]. Test method for: - Infants < 1 year of age, PCR or culture - Individuals > 1 year of age and with > 3 weeks of coughing, serology^b^ - Individuals > 1 year of age and with < 3 weeks of coughing, PCR [24,40,45].
	Medical treatment of index case and/or at-risk contacts	First confirm the clinical diagnoses of the index case by laboratory test. In a possible index case whose family includes unvaccinated or incompletely vaccinated infants < 1 year of age, a woman > 34 weeks pregnant or a child with severe heart or lung failure, treatment is indicated for all family members and can start before laboratory confirmation of the index case. Medical treatment outside the family only occurs after PHS advice and laboratory confirmation of the index case [24,45]. Preferably, start treatment of the index case within 3 weeks of illness onset [24,45].
	Vaccination of at-risk contacts	Administer first vaccination prior to vaccination of NIP or administer vaccination to unvaccinated or incompletely vaccinated children < 5 years old in the family [24,45].
	Notification of local PHS [23]	Patients with typical symptom(s)^a^ or with at least 14 days of coughing combined with either a positive laboratory test or contact within past three weeks with a confirmed pertussis case [24]. Notify within one workday [44].
**Laboratory**	Laboratory diagnostics	Interpret as positive for pertussis when detection of *B. pertussis* or *B. parapertussis* or high antibody titre in single serology^b^ or significant increase of titre in multiple serology.
	Notification of local PHS [23]	Patients with typical symptom(s)^a^ or with at least 14 days of coughing combined with either a positive laboratory test or contact within past three weeks with a confirmed pertussis case [24]. Notify within one workday [44].
**Local public health services**	Surveillance	Collect notifications and clinical data from HCPs and laboratories and report it to RIVM [23]. Notify within one week [44].
	Medical treatment and/or vaccination advice to the patient’s HCP	Provide advice on medical treatment and vaccination according to national guidelines.

HCP: healthcare provider; NIP: National Immunisation Programme; PHS: public health services; RIVM: National Institute for Public Health and the Environment.

^a^ Typical symptoms include paroxysmal coughing, a whooping sound after coughing or vomiting after coughing [24].

^b^ Single serological testing is not suitable to detect recent infection in individuals vaccinated with an acellular pertussis vaccine within the past year. Multiple serology is also recommended when the first titre is below the cut-off value specific for a pertussis infection [24].

Pertussis surveillance aims to monitor the impact of the vaccination programme, identify high-risk areas and detect outbreaks, monitor case management and take timely preventive measures [25]. However, pertussis surveillance and control are greatly hampered by, for example, under-ascertainment as individuals with mild symptoms or who are asymptomatic may not present to healthcare for diagnosis [26,27]. Clinically, the disease resembles other respiratory diseases, particularly in the early catarrhal phase when an individual is already highly contagious [26]. Also, the classical pertussis symptoms are often absent in adolescents and adults such that these cases may not be recognised by HCPs [26,28,29]. In 2006–2007, the estimated seroprevalence of pertussis infections was 100-fold higher than the reported notifications at that time [15]. Laboratory diagnostic procedures, especially interpretation of serology, are complicated because of changing cut-off value recommendations, in cases of recent immunisation and the limitations of available diagnostic tests [5,27,30]. As the pertussis diagnostic process is quite challenging, not all cases presenting to healthcare are diagnosed (underdiagnosis) or notified (under-notification) [31]. Moreover, there is debate about how to reduce the overall delay until notification to improve pertussis control [32,33].

A quantification of healthcare and public health factors that may compromise the accuracy of pertussis surveillance and quality of pertussis control in infants < 1 year of age in day-to-day practice is missing. This study aims to assess possible pertussis misdiagnosis, under-notification and delay in laboratory testing and delay in notifications to PHS.

## Methods

We evaluated the possible pertussis misdiagnosis, under-notification and delay using laboratory testing data and notification data from local PHS.

### Study location

This study was conducted in Limburg, the southern-most province of the Netherlands. There are 1.1 million inhabitants and this population is comparable to the rest of the Netherlands in terms of sex composition and urbanisation, although it is slightly older [34]. All six medical microbiology laboratories in this province provided test data for this study. Notification data were retrieved from the two local PHS.

### Laboratory records and standardisation of results

Laboratory data from pertussis testing requested by HCPs (general practitioners (GPs) and hospital specialists) and conducted by all laboratories in the study area between 2010 and 2013 were retrieved. The data included patient data on age, type of test performed (serology IgA/IgG, PCR and culture), type of HCP (GP or specialist), date of test request, date of test result, test result and interpretation of test result. No data on sex was reported. Based on regional GP testing-behaviour questionnaires, we estimated that the laboratory data covered at least 90% of all pertussis tests in our region. A total of 12,090 tests on 10,131 individuals were performed.

#### Laboratory tests

The laboratories used different test assays, cut-off values and algorithms for interpreting test results, including interpretation of disease duration and patient age as the sensitivity of the diagnostic tests for pertussis depends on the age of the patient and the duration of illness [35]. Whether laboratories received clinical data to include into their algorithms depends on the HCP who requested the test. *B. pertussis* IgA and/or pertussis toxin IgG ELISA from Virion/Serion (Würzburg, Germany) or Virotech (Rüsselsheim, Germany) was used for serology. The sensitivity and specificity of these serological test kits varied between 68% and 89% and between 67% and 87%, respectively [36]. At one laboratory, the serological cut-off values changed on 1 September 2011. Two laboratories switched from Virotech Units (VU) to International Units (IU), one on 1 May 2011 and the other on 16 January 2012. Charcoal blood agar (Oxoid) with and without cephalexin were used for culture. Four of the laboratories used an in-house multiplex PCR test with target gene *IS481* (*B. pertussis*) and target gene *IS1001* (*B. parapertussis*). The other two laboratories were unable to distinguish between *B. pertussis* and *B. parapertussis* because target gene *IS1002* or target gene *IS481* were used.

#### Serology interpretation

There are a lack of uniform laboratory guidelines on serology interpretation so different cut-off values are used by laboratories across Europe [27,37]. To correct for the different cut-off values used in our study area and to detect other inter-laboratory differences, we compared laboratory test interpretation to standardised serological test results. The conversion from VU/ml to IU/ml was calculated with the following formula: $\left({\left(number\;of\;VU/ml\;/\;\left(33.3-number\;of\;VU/ml\right)\right)}^{1.15}\right)\;\times \;112=\;IU/ml$.

A single high titre of IgG ≥ 62.5 IU/ml or IgG ≥ 13 VU/ml was defined as positive as these cut-off values have been shown to be sensitive and specific indicators of infection in the past year [15,38,39]. International guidelines recommend measuring IgA antibodies with intermediate IgG levels or when no second sample can be obtained [40,41]. IgA antibodies were not taken into account in our standardisation as most laboratories did not use IgA antibody results in their serology interpretation. Furthermore, measuring IgA antibodies has been proven to be less specific and sensitive [5,36]. The standardised test result was considered positive when multiple serology was applied and all serology tests were positive, and it was considered negative when all serology tests applied were negative. When multiple serology test results were inconsistent, the standardised test result was considered positive when seroconversion occurred from a negative test to a positive test result.

#### Notification data

Notification data were collected from the two local PHS that serve the study area. These data included date of first day of illness, notifier (laboratory/GP/hospital), date of notification to PHS, date of notification to national notification system of RIVM and information on preventive measures taken by the PHS, including giving advice and providing vaccination or prophylaxes to at-risk contacts.

### Statistical analyses

The laboratory data were analysed at the test-level and at the episode-level. Notably, a possible episode of pertussis disease was considered unique if the same individual was tested once or more within an 8-week interval. However, if an individual was tested twice within an interval longer than 8-weeks it was considered two possible pertussis episodes. Descriptive statistics and chi-squared tests were used to compare categorical variables such as type of HCP, laboratory, type of test and year of test.

Independent sample t-tests were performed to study differences in delay. For analyses of the notification data, we used descriptive statistics. Analyses were performed using the SPSS package version 21.0 (IBM Inc., Somers, New York, United States).

## Results

### Possible misdiagnosis (underdiagnosis and overdiagnosis) of pertussis

Of all HCP-requested pertussis tests done in the study region in 2010–2013 (n = 12,090), the majority (81%, n = 9,818) were requested by GPs, varying from 72% to 88% per laboratory (p < 0.001). The remaining pertussis tests were requested by hospital specialists (19%, n = 2,272). Most tests (93%, n = 11,190) were serological tests, 6% (n = 729) were PCR tests and culture was performed in 1% (n = 171). This distribution of test type differed between laboratories, ranging from, for example, 77% to 99% for serological tests (p < 0.001). Serological tests were more likely to be requested by GPs (95%, n = 9,275) compared with hospital specialists (84%, n = 1,915), p < 0.001.

In 44% (134/303) of tested infants < 1 year of age, serology was performed instead of the recommended PCR or culture (Table 2). This proportion differed by laboratory, varying from 24% to 70% (p < 0.001) and decreased over time, from 64% in 2010 to 33% in 2013 (p < 0.01). In these infants, GPs requested serology more often (65%, 52/80) than hospital specialists (37%, 82/223), p < 0.001.

**Table 2:** Recommended tests and performed serology pertussis testing stratified into age groups, the Netherlands, 2010–2013

Age group	Recommended test [24,40,45]	Total number of performed tests (n = 12,090)	Number of performed serology tests (n = 11,190)	Percent of performed serology tests (%)
**< 1 year**	PCR or culture	303	134	44
**> 1 year with > 3 weeks of coughing**	Serology	11,787	11,056	94
**> 1 year with < 3 weeks of coughing**	PCR			

**Figure 1:**
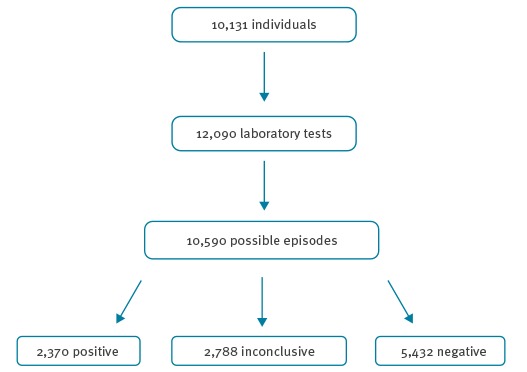
Possible pertussis episodes^a^ considered positive, inconclusive and negative by laboratories^b^, the Netherlands, 2010–2013 (n = 12,090)

^a^ A possible episode of pertussis disease was considered unique if the same person was tested once or more within an 8-week interval. If an individual was tested twice within an interval longer than 8-weeks, it was considered two possible pertussis episodes.

^b^ The laboratories used different test assays, cut-off values and algorithms for interpreting serology, PCR and culture test results as positive, inconclusive or negative.

In total, 10,590 possible pertussis episodes were identified between 2010 and 2013 (Figure 1). Overall, 22% (n = 2,370) of these possible episodes were interpreted by the laboratory as positive, with this varying between laboratories (15% to 28%) and over time (from 11% in 2010 to 27% in 2012) without a clear trend, p < 0.001. Serological tests had higher positivity rates (23%, 2,276/9,736) compared to PCR tests and culture with positivity rates of 12% (83/715) and 8% (11/139) respectively, p <0.001.

Furthermore, in 26% (n = 2,788) of the possible episodes, no conclusive laboratory test interpretation was available. These tests were inconclusive due to missing additional serological testing (n = 1,520), missing clinical data such as the first day of illness (n = 321), the possibility of past infections or antibodies after vaccination (n = 214) and no/dubious result available (n = 733). The proportion of possible episodes with an inconclusive laboratory result varied between laboratories (0% to 60%) and increased over time (from 18% in 2010 to 35% in 2013), p < 0.001.

Of all possible pertussis episodes with an available IgG-titre (n = 8,929), 22% (n = 1,998) were positive according to the laboratory and 19% (n = 1,700) appeared positive after standardisation. This standardised positivity rate varied between laboratories (15% to 23%) and over time (9% in 2013 to 24% in 2012), p < 0.001. Of all episodes considered positive by the laboratory, 32% (n = 644) were negative after standardisation. This was due to high IgA-titres in combination with IgG-titres below 62.5 IU/ml. Of all inconclusive episodes, 9% (n = 248) were positive after standardisation. This was due to the use of a grey area in both the interpretation of IgA- and IgG-titres of some laboratories in combination with IgG-titres higher or equal to 62.5 IU/ml. Of all negative episodes, 2% (n = 98) were positive after standardisation (Table 3).

**Table 3:** Standardised test results for possible pertussis episodes using serology with available IgG-titres, Limburg province, the Netherlands, 2010–2013

Laboratory interpretation of possible episodes with IgG titres (n = 8,929)	Standardised^a^ result negative (n = 7,229)			Standardised^a^ result positive (n = 1,700)		
	Number (n)	Percent (%)	Variation between laboratories (range of %)	Number (n)	Percent (%)	Variation between laboratories (range of %)
**Positive (n = 1,998)**	644	32	0–57^b^	1,354	68	43–100^b^
**Inconclusive (n = 2,672)**	2,424	91	45–95^c^	248	9	5–55^c^
**Negative (n = 4,259)**	4,161	98	90–100	98	2	0–10

^a^ Cut-off values of IgG ≥ 62.5 IU/ml and IgG ≥ 13 VU/ml were used for standardisation. A single high titre at/above these values was defined as positive. The standardised test result was considered positive when multiple serology was applied and all serology tests were positive, and it was considered negative when all serology tests applied were negative. When multiple serology test results were inconsistent, the standardised test result was considered positive when seroconversion occurred from a negative test to a positive test result.

^b^ p < 0.01.

^c^ p < 0.001.

### Under-notification of pertussis

Of all notifications to local PHS between 2010 and 2013 (n = 2,241), 93% (n = 2,090) were notified by a laboratory. The remaining notifications were by a GP (3%), a hospital (2%) or others (2%). Of the total number of laboratory-positive episodes of persons living in the study region (n = 2,301), 412 (18%) were not notified to the PHS. This under-notification varied between laboratories from 10% to 39%, and varied from 13% in 2011 to 59% in 2010 with no trend, p < 0.001. All notifications were evaluated and verified by the local PHS, which were only able to take timely preventive measures, such as giving advice or providing vaccination or prophylaxes to at-risk contacts, in 1% (n = 26) of all notifications.

### Delay in pertussis control cascade

**Figure 2:**
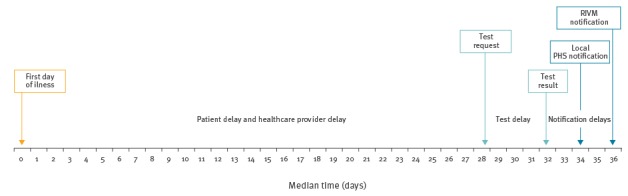
Median time of *Bordetella pertussis* and *Bordetella parapertussis* infection from first day of illness to notification of the RIVM, Limburg province, the Netherlands, 2010–2013

PHS: public health services; RIVM: National Institute for Public Health and the Environment.

The median time between patients’ first day of illness and HCP request for a laboratory test, patient and HCP delay, was 28 days (interquartile range (IQR): 21–47) (Figure 2). It was longer for serological test requests (median = 29 days, IQR: 21–49) compared with PCR or cultures (median = 18 days, IQR: 13–24), p < 0.001. For infants, this delay was shorter (median = 12 days, IQR: 6–19) compared with all other ages (median = 28 days, IQR: 21–48), p < 0.05. Of all laboratory notifications, 28% (n = 571) were tested within 3 weeks. Median time from a requested laboratory test to a test result, median test delay, was 4 days (IQR: 3–7). The median test delay was longest for culture (7 days) and shortest for PCR and serology (4 days), p < 0.001. In terms of notification delay, the median time between patients’ first day of illness to local PHS notification was 34 days (IQR: 27–54). Of all laboratory notifications, 12% (n = 245) were notified to the local PHS within 3 weeks. It then took local PHS a median of 2 days (IQR: 0–5) to collect all patient and patient contacts information for a proper risk assessment and to notify RIVM. This is usually done by contacting HCPs for further essential risk information in order to decide on any necessary preventive measures. In 96% (1,538/1,601) of the notifications, it took less than 7 days for the local PHS report to the national system of RIVM.

## Discussion

This study reveals possible pertussis misdiagnosis by both HCPs and laboratories, substantial under-notification of positive pertussis episodes by laboratories, and a large delay in the pertussis control cascade. All these factors negatively impact control strategies and jeopardise the effectiveness of the national pertussis surveillance system. The accuracy of pertussis surveillance is of urgent interest to all countries using notification data to guide pertussis surveillance and control. In our study, large variations in test behaviour, laboratory assays used, test interpretation and notification behaviour were observed between HCPs and between laboratories in the pertussis diagnostic process. This variation is likely to cause the accuracy of surveillance data to be different at the local level, yet which areas are more accurate than other areas is unknown. These results are also likely applicable to the other countries in Europe with similar surveillance systems considering the generalisability of misdiagnosis, under-notification and overall delay in surveillance data.

Just slightly over half of the infants were tested for pertussis using the recommended PCR or culture. Furthermore, a quarter of possible episodes in all ages lacked a conclusive laboratory test result. These results show the possibility of misdiagnosis and the complexity of the pertussis diagnostic process both for HCPs and for the laboratories. It is unknown whether the possible misdiagnoses caused underdiagnosis or overdiagnosis. The standardisation of test results using IgG-titres revealed that positivity rate differences between laboratories remained after correcting for the different cut-off values used. Laboratory differences in test interpretation, the variation in tests used and positivity rates have also been found in prior studies [5,27,30].

Our study shows that almost one fifth of all laboratory pertussis diagnoses were not notified to the local PHS. A comparable under-notification rate has been reported in Italy [42]. Administrative and logistical problems are possible contributors to this under-notification. In the laboratory that used an automatic digital notification system, there was less under-notification (10%) compared to the other laboratories, p < 0.001.

There was a considerable delay in the national surveillance data. Overall, it took a median of 34 days from first day of illness before the local PHS was notified, and only 12% of all laboratory notifications were reported within three weeks, which is comparable to other Dutch findings [32,43]. Time from the laboratory test result to local PHS notification and from local PHS to RIVM was in accordance with the guidelines [44]. However, time from the first day of illness to a laboratory test request was 28 days, while pertussis remains contagious up to 3–4 weeks after the first symptoms [5]. Clinical diagnosis and diagnosis after laboratory testing is therefore made too late to start treatment or take any necessary preventive measures. Adequate and early diagnosis followed by antibiotic treatment is particularly important as it can prevent further transmission to infants, HCPs and pregnant women [21]. For optimal effectiveness, treatment has to be started early after onset of illness as pertussis is no longer present in respiratory secretions after about 3 weeks [45]. The long patient and/or HCP delay seen in this study therefore limits early treatment and optimal pertussis control.

This study benefited from a large sample size and having complete regional data via a large database that included around 90% of laboratory tests for pertussis in one geographical area. Another strength was the availability of information on advice given or preventive measures taken by the local PHS for all notifications. Moreover, we studied data from across the pertussis control chain for both healthcare as well as public health. However, data on patient symptoms, disease awareness or healthcare-seeking behaviour were lacking. We were therefore unable to assess these patient-related factors as an explanation for patient delay. More information on the HCP’s reasons to test for pertussis would have been desirable. It would be of interest to know whether they initiated any preventive measures for close contacts since notification often comes too late for local PHS to take action. Given that date of consultation was not known, we were unable to identify how much delay was attributable to patient and HCP delay, respectively. We were also unable to estimate under-notifications specifically for notifications by GPs or hospital specialists since we have no data on the number of clinical diagnoses. At last, the standardisation of laboratory results was only based on IgG antibodies and did not take any clinical data like vaccination status, duration of coughing or age into account. This meant that we were not able to estimate possible false positives or false negatives, but this limited standardisation does illustrate differences between laboratories that could lead to local differences in diagnosis or misdiagnosis.

## Conclusion and recommendations

In conclusion, this study revealed several factors that prevent good pertussis control by PHS by contributing to misdiagnosis, under-notification and delay in notifications. These factors include suboptimal testing behaviour, laboratory diagnostic procedures, and notification behaviour. While the number of notifications are the current basis for pertussis surveillance, the accuracy of this indicator for disease occurrence and as a management tool is likely poor.

The accuracy of surveillance would be improved by focusing on the factors identified here. First, to reduce misdiagnosis and the variation in pertussis diagnostics, we recommend that laboratories and HCPs improve their adherence to national guidelines about when to perform which type of test and on whom. A national uniform guideline on serology cut-off values and the use of IgA and/or IgG is desirable. Testing all patients presenting with cough is not feasible as previous research has shown that only 3% of adult patients in 12 European countries presenting with acute cough in primary care had evidence of an acute pertussis infection [46]. In the Netherlands, the current pertussis incidence is largely the result of testing and more testing would not necessarily improve pertussis control [47]. Therefore, HCPs should focus more on diagnosing patients with pertussis-like symptoms who have pregnant women or infants in their proximity. Second, to reduce under-notification, laboratories and HCPs could benefit from using an automated notification system. Third, as public health is almost always too late to intervene, preventive measures should be carried out earlier in the pertussis control chain of actions. GPs, midwives and child care workers could play a major role here. Creating awareness among these professionals and patients about taking timely preventive measures could lead to lower individual disease burden and increased pertussis care cost-effectiveness. Additional preventive measures such as the recommended maternal vaccination [18] and shortening the chain of actions would contribute to improving the surveillance system and more importantly, preventing pertussis infection, morbidity and mortality prevention in infants.
